# Relationship between physical literacy and mental health in adolescents: a moderated mediation model with resilience and physical activity as variables

**DOI:** 10.3389/fpsyg.2025.1518423

**Published:** 2025-02-04

**Authors:** Zhu Shanshan, Tao Ping, Lin Jiabin, Liu Tianzhuo, Lai Xiaomei, Wang Bolei, Dai Leifu, Tang Jianfeng

**Affiliations:** ^1^School of Physical Education, Northeast Normal University, Changchun, Jilin, China; ^2^School of Physical Education, Changchun Normal University, Changchun, China; ^3^College of Sports Science, Shenyang Normal University, Shenyang, Liaoning, China

**Keywords:** physical literacy, mental health, physical activity, resilience, adolescence

## Abstract

**Introduction:**

Physical literacy is defined as the motivation, confidence, physical competence, knowledge, and understanding to value and take responsibility for engaging in physical activities for life, and may be associated with higher mental health; however, few studies have explored the processes underlying these positive associations.

**Objectives:**

The present study aimed to explore the role of resilience in the relationship between physical literacy and mental health, and further examines the mediating role of physical activity.

**Methods:**

This cross-sectional study recruited a sample of 1,022 (aged 10–18 years, Mage = 14.84, SD = 1.79, 44.9% male) Chinese students, grades five to twelve, via physical education courses. The hypothesized moderated mediation model was employed using Model 4 and Model 8 of the PROCESS macro for SPSS to examine the relationships between physical literacy, mental health, resilience, and physical activity.

**Results:**

Physical literacy was positively and significantly correlated with mental health (*r* = 0.364, *p* < 0.001), and resilience (*r* = 0.486, *p* < 0.001). After controlling for sex and grade variables, resilience mediated the relationship between adolescent physical literacy and mental health, physical activity moderated the relationship between physical literacy and resilience in the mediator model but not between physical literacy and mental health.

**Discussion:**

The present study suggests that could physical literacy predict increased levels of resilience, which could then lead to higher mental health. However, this mediation effect may fluctuate across individuals with different levels of physical activity. Overall, this study may reveal that physical literacy promotes resilience and mental health processes in individuals with different levels of physical activity.

## 1 Introduction

Physical literacy has become a vital topic in sports-related professions, including physical activity, physical education, etc. (Belanger et al., 2018; Brown et al., 2020). Physical literacy is a multidimensional concept that reflects ongoing changes and integrates cognitive, psychological, social, and physical capabilities (Barnett et al., 2023). Physically literate individuals are described as having the knowledge, confidence, motivation, physical competence, and understanding to respect and accept responsibility for participating in physical activities throughout life (IPLA, 2017). The concept is rooted in existential and phenomenological philosophy, which describes an embodied structure seeking harmony and unity between physical, spiritual, and environmental states (Whitehead, 2019), and is becoming more widely recognized as a vital human capacity owing to its synergy with human prosperity (Durden-Myers et al., 2018). Consequently, a growing body of research suggests a positive relationship between physical literacy, physical fitness (Caldwell et al., 2020; Pastor-Cisneros et al., 2021), and physical activity levels (Ma et al., 2020b; McKay et al., 2023). Most previous studies focused on associating physical literacy with physical domains, aiming to positively influence strategies that encourage engagement in physical activity through physical literacy (Ma et al., 2021). However, little is known how physical literacy could affect resilience and mental health, and studies that combine physical literacy, mental health, and physical activity have not attracted much attention. Thus, the present study aimed to explore the relationship between physical literacy, resilience, mental health, and physical activity.

### 1.1 Physical literacy and mental health

Mental health is critical to an individual's comprehensive wellness and is as essential as physical health, and is defined as a state of wellbeing in which individuals realize their own potential, can cope with the normal stresses of life, can work productively and fruitfully, and contribute to their community (WHO, 2021a,b). Commonalities exist between mental health and physical literacy. First, both are closely related to human flourishing. Mental health encompasses both the absence of emotional turmoil, such as anxiety, loneliness, and depression (referred to as “emotional problems”), and positive indicators of health, including vitality, life satisfaction, and prosperity (Topp et al., 2015; Van de Casteele et al., 2024). Globally, physical literacy is gradually gaining acceptance owing to its contribution to human prosperity (Durden-Myers et al., 2018). Second, mental health is considered a state of overall cognitive, behavioral, and affective wellbeing (Espie et al., 2019). Similarly, based on Whitehead's (2019) theoretical model, the emotional, physical, and cognitive domains form the foundation of physical literacy development. Although physical literacy and mental health may be related owing to shared traits and skills that enhance individuals' wellness and life quality (Ma et al., 2021), few studies linked physical literacy with mental health. A study using structural equation models revealed that physical literacy was positively associated with vitality, positive emotions, recreational physical activity, and engagement in physical education (PE) lessons but inversely related to negative emotions (Blain et al., 2021). However, the mechanism through which physical literacy affects mental health remains unclear, emphasizing the need for comprehensive research to examine the association between the two, and the role of mediators and moderators of this relationship.

### 1.2 The mediating role of resilience

Resilience refers to one's ability to positively adapt and respond to adversity through interactions with the surrounding environment (Jefferies et al., 2019; Rutter, 2012). Adolescents' ability to thrive under adverse circumstances depends on their connection with all facets of their surroundings and the extent to which these surroundings can increase or preserve optimal psychological, social, and physical health (Masten, 2014; Meng et al., 2023). This definition corresponds with physical literacy, which advocates interaction with one's environment to enhance the physical and social environment (Dudley et al., 2017). When an individual is confronted with real-life stressors, the key factors of physical fitness and resilience are improved (Ma et al., 2021). Furthermore, the synergistic relationship between physical literacy and resilience becomes apparent when an environment is established to help develop the ability to overcome challenges, obstacles, and adversity (Meng et al., 2023). Jefferies et al. (2019) reported that physical literacy is positively associated with resilience. Moreover, resilience can act as an intermediary between other related factors and mental health (Yildirim et al., 2020; Zhao et al., 2020). A study on 5,835 undergraduates revealed the mediating role of resilience between physical fitness and mental health (Ma et al., 2021). Additionally, resilience acts as a mediating variable between physical literacy and psychological wellbeing in adults aged 25–48 (Meng et al., 2023). However, to the best of our knowledge, no previous studies examined the relationships among physical literacy, resilience, and mental health in adolescents. Given that adolescents are particularly vulnerable to mental health deterioration, this is a significant field of research.

### 1.3 The moderating role of physical activity

The relationships between resilience, mental health, and physical literacy may be conditioned by several factors associated with individual differences. Among these factors, physical activity level is often highlighted as a variable that can modify the level of physical literacy, resilience, and mental health. Brown et al. (2020) found that after controlling for sex and socioeconomic status, physical activity engagement was the highest among individuals with high physical literacy profiles, followed by those with moderate and low physical literacy profiles. A cross-lagged analysis revealed that physical activity as a causative variable significantly impacted resilience in adolescents (Guo and Liang, 2023). The results also indicated that individuals who continuously engaged in routine physical activity exhibited greater resilience than those who did not; however, no variations in mental health were observed (Wermelinger Ávila et al., 2022). Nevertheless, physical activity remains an important mental health intervention (DeJonge et al., 2020; White et al., 2017; Wilski et al., 2024). Therefore, this study speculated that physical activity may moderate the relationship between physical literacy, mental health, and resilience.

### 1.4 The present study

Extant studies increasingly support the beneficial relationship between mental health and physical literacy. However, limited information is available concerning the variables that may affect this relationship, such as resilience and physical activity.

Based on the available findings, it seems possible to speculate that mental resilience can be predicted by physical literacy and can predict mental health. Namely, resilience could mediate the relationship between physical literacy and mental health. Moreover, for individuals with different level of physical activity, the effects of physical literacy on resilience and mental health may be different. Therefore, based on previous study findings, this study examined whether adolescents' physical literacy is associated with their mental health and the extent to which resilience mediates this relationship, considering physical activity level as a moderating factor.

**Figure 1:**
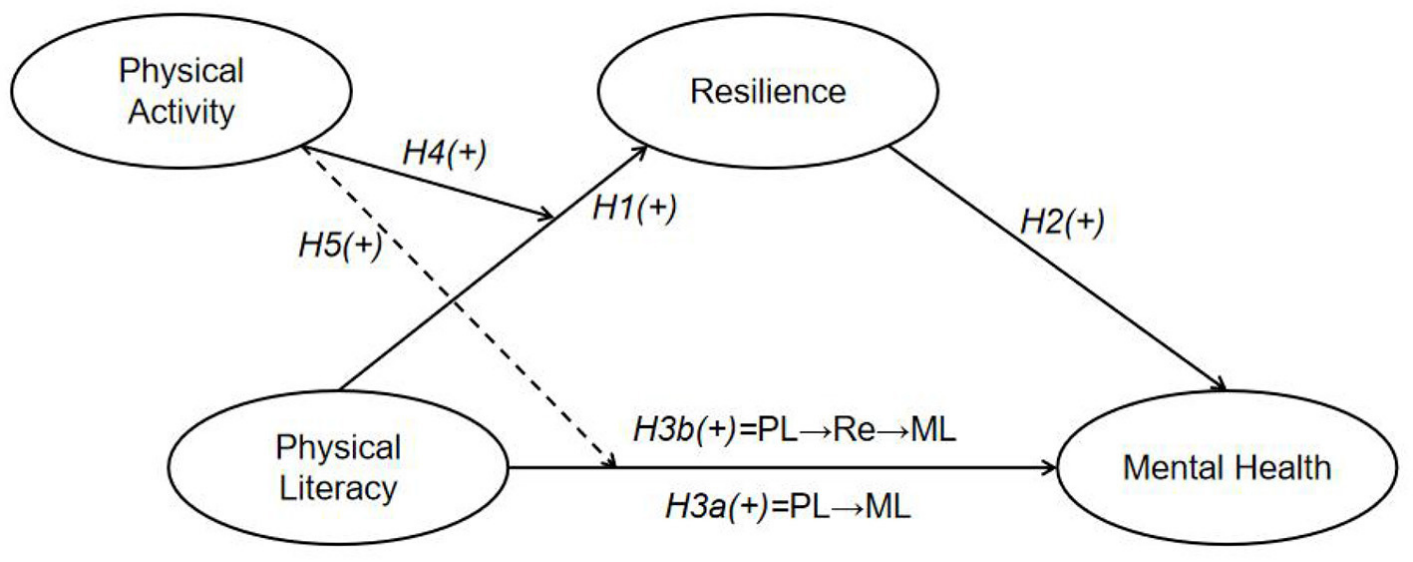
Conceptual framework.

Accordingly, this study presented two questions: Does resilience play a mediating role between mental health and physical literacy in adolescents? and does the mediating effect of resilience on the relationship depend on physical activity? Subsequently, this study proposed five hypotheses (Figure 1 presents the conceptual framework):

1. Hypothesis 1 (H1): physical literacy positively influences adolescents' resilience.
2. Hypothesis 2 (H2): resilience positively influences adolescents' mental health.
3. Hypothesis 3 (H3a): physical literacy positively influences adolescents' mental health and (H3b) resilience mediates the relationship between physical literacy and mental health in adolescents.
4. Hypothesis 4 (H4): physical activity moderates the relationship between physical literacy and resilience.
5. Hypothesis 5 (H5): physical activity moderates the relationship between physical literacy and mental health.

## 2 Materials and methods

### 2.1 Participants and procedure

This cross-sectional study was conducted using a random cluster sample of primary and secondary school students from November 1 to December 10, 2023. Eligible fifth to twelfth graders from four different schools in Changchun, Jilin Province, China, who completed physical education courses, were recruited by four teachers after obtaining approval from the school principals and the students' parents. Confidentiality was maintained at all times during the survey process and participants had the right to opt out at any time. All procedures were carried out in accordance with the standards of the Ethics Committee of Northeast Normal University, the 1964 Helsinki Declaration, and its later amendments, and all participants provided written informed consent before participating. The questionnaire was administered after the minor participants obtained written consent from their guardian class director at the school.

The sample size comprised 1,176 primary and secondary school students. After excluding participants who provided incomplete data, answered with regularity, or failed to pass the lie testing item, the valid data of 1,022 participants [Mage = 14.84, standard deviation (SD) = 1.79, 44.9% male, aged 10–18 years] were included.

### 2.2 Measures

#### 2.2.1 Physical literacy

Students' physical literacy was evaluated using the simplified Chinese version of the perceived physical literacy instrument (PPLI; Sum et al., 2016). The PPLI utilized in this study was derived from the original Cantonese version employed by PE teachers in Hong Kong. Sum et al. (2018) trimmed the original 18-item scale into nine items and used it to measure the physical literacy of Hong Kong adolescents (Ma et al., 2020a; Sum et al., 2018), which includes three dimensions (“sense of self and self-confidence,” “self-expression and communication with others,” and “knowledge and understanding”), each containing three items through exploratory factor analysis. Each item was rated on a five-point Likert scale ranging from 1 (*strongly disagree*) to 5 (*strongly agree*), with an overall score ranging from 9 to 45. The simplified Chinese version of the PPLI exhibited excellent retest reliability and construct validity (Xin, 2020).

#### 2.2.2 Physical activity

Students' physical activity was evaluated using the revised version of the Physical Activity Rate Scale, known as PARS-3 (Liang and Liu, 1994). This scale evaluates the extent of exercise based on time, intensity, and frequency of participation rated on a five-level scale from 1 to 5, where exercise volume = intensity × (Time − 1) × frequency (Lin and Zhu, 2022; Lin et al., 2024). The maximum and minimum scores are 100 and 0, respectively. The evaluation criteria for the exercise volume were as follows: ≤ 19 points = low exercise volume, 20–42 points = moderate exercise volume, and ≥43 points = high exercise volume (Jiang and Zhu, 1997). The internal consistency was 0.856 and the retest reliability was 0.82 (Ding et al., 2016).

#### 2.2.3 Resilience

Students' resilience levels were measured using the Brief Resilience Scale (BRS) compiled by Smith et al. (2008). Each of the scale's six items (three forward and three reverse items) was rated on a five-point Likert scale ranging from 1 (*strongly disagree*) to 5 (*strongly agree*). The BRS is reliable for evaluating resilience to stress (Rodríguez-Rey et al., 2016; Sánchez et al., 2021; Smith et al., 2008). Moreover, the Chinese version of the BRS demonstrated good validity and reliability in measuring psychological resilience (Chen et al., 2020; Tian, 2021).

#### 2.2.4 Mental health

To assess the mental health status of the adolescents in this study, a single self-rated item was used (“How would you rate your current mental health state [stress, sadness, emotional challenges, etc.]?”). Participants responded on a five-point Likert scale ranging from 1 (*very poor*) to 5 (*excellent*).

### 2.3 Statistical analysis

Regarding the mediation analysis of the quantitative data, SPSS (Version 26.0; IBM) and the PROCESS macro were utilized. The statistical analyses involved several steps. First, preliminary analyses of the study variables were conducted to assess normality, multicollinearity, and common method bias (MacKenzie and Podsakoff, 2012; Lin et al., 2024). Second, descriptive statistics were used to examine differences in the primary variables based on sex and age using *t-*tests and analysis of variance. Third, after adjusting for one-child status, gender, and age, partial correlation analyses were conducted to determine the relationships between resilience, mental health, physical activity, and physical literacy. Finally, a moderated mediation effect analysis was performed to determine whether the relationship between mental health and physical literacy was mediated by resilience and whether physical activity served as a mediator. The indirect effects were estimated using standard errors and a 95% confidence interval (CI; Hayes and Rockwood, 2017). The significance level for all statistical analyses in this study was set at *p* < 0.05 (two-sided).

## 3 Results

### 3.1 Preliminary analyses

In instances of missing data in the physical literacy, resilience, physical activity, and mental health scales, multiple imputations were employed. Screening of normality statistics (e.g., skew and kurtosis) revealed no problematic distributions of quantitative items and no multicollinearity issues among the study variables. Furthermore, factor analysis of all measurement indices (resilience, mental health, physical activity, and physical literacy) revealed that the initial eigenvalue of the first factor was 37.57%. This indicated the lack of significant common method bias, given that the value did not exceed 40%.

#### 3.1.1 Descriptive statistics

Table 1 presents the descriptive statistics for the main variables. Among the 1,022 adolescents (459 males, 44.9%), the mean scores for physical literacy, physical activity, resilience, and mental health were 34.45 (SD = 6.68), 20.21 (SD = 20.76), 20.51 (SD = 4.78), and 3.73 (SD = 1.08), respectively. Notably, significant variations were observed among some demographic characteristics. Most variables, including age, physical literacy (overall, knowledge and understanding, self-expression and communication with others, except for sense of self and self-confidence), physical activity, resilience, and mental health, exhibited notable differences between males and females. Specifically, males demonstrated significantly higher scores than females across all variables except for sense of self and self-confidence. Regarding physical activity levels, 61.3% (*N* = 625) of participants reported a low exercise level, while 24.9% (*N* = 254) and 13.7% (*N* = 140) reported moderate and high levels, respectively.

**Table 1:** Descriptive statistics by sex.

**Variables**	**Total (*N =* 1,022) M ±SD**	**Male (*N =* 459) M ±SD**	**Female (*N =* 563) M ±SD**	***t***	***p***
Age (years)	14.84 ± 1.79	14.98 ± 1.81	14.73 ± 1.76	2.218^*^	0.027
PL	34.45 ± 6.68	35.32 ± 6.81	33.73 ± 6.48	3.798^**^	0.000
SSSC	11.47 ± 2.90	11.62 ± 2.74	11.34 ± 3.02	1.530	0.126
SEC	10.68 ± 2.81	11.00 ± 2.91	10.41 ± 2.70	3.356^**^	0.001
KU	12.30 ± 2.37	12.70 ± 2.43	11.98 ± 2.27	4.843^**^	0.000
PA	20.21 ± 20.76	26.39 ± 23.22	15.18 ± 16.95	8.620^**^	0.000
RE	3.41 ± 0.81	3.60 ± 0.78	3.26 ± 0.78	6.754^**^	0.000
MH	3.73 ± 1.08	3.81 ± 1.06	3.66 ± 1.09	2.178^*^	0.030

PL, physical literacy; SSSC, sense of self and self-confidence; SEC, self-expression and communication with others; KU, knowledge and understanding; PA, physical activity; RE, resilience; MH, mental health. ^*^p < 0.05, ^**^p < 0.001.

#### 3.1.2 Correlations of all tested variables

The relationship between variables was assessed using Pearson's correlation coefficient after controlling for sex and age. Table 2 presents the correlation coefficients. The results revealed that mental health was positively correlated with physical literacy (*r* = 0.364, *p* < 0.001), sense of self and self-confidence (*r* = 0.323, *p* < 0.001), self-expression and communication with others (*r* = 0.329, *p* < 0.001), knowledge and understanding (*r* = 0.238, *p* < 0.001), and resilience (*r* = 0.439, *p* < 0.001). However, the correlation between mental health and physical activity was not significant (*r* = 0.012, *p* > 0.05).

**Table 2:** Correlations matrix among tested variables.

**Variables**	**PL**	**SSSC**	**SEC**	**KU**	**PA**	**RE**	**MH**
PL	1						
SSSC	0.859^**^	1					
SEC	0.816^**^	0.534^**^	1				
KU	0.790^**^	0.556^**^	0.453^**^	1			
PA	0.325^**^	0.237^**^	0.244^**^	0.335^**^	1		
RE	0.486^**^	0.366^**^	0.502^**^	0.322^**^	0.156^**^	1	
MH	0.364^**^	0.323^**^	0.329^**^	0.238^**^	0.012	0.439^**^	1

PL, physical literacy; SSSC, sense of self and self-confidence; SEC, self-expression and communication with others; KU, knowledge and understanding; PA, physical activity; RE, resilience; MH, mental health. ^**^p < 0.001.

### 3.2 Mediating effect analysis

Model 4 in the SPSS PROCESS macro (Version 26.0; IBM) was used to examine how resilience mediated the relationship between mental health and physical literacy (Hayes, 2013), after controlling for sex and age. The findings indicated that the regression analyses of physical literacy to mental health [*c* = 0.179, SE = 0.014, 95% CI = (0.151, 0.207)], physical literacy to resilience [*a* = 0.17, SE = 0.01, 95% CI = (0.153, 0.191)], physical literacy to mental health [*c*' = 0.097, SE = 0.015, 95% CI = (0.067, 0.128)], and resilience to mental health [*b* = 0.472, SE = 0.044, 95% CI = (0.387, 0.558)] were significant. Therefore, resilience partially mediated the relationship between physical literacy and mental health. The mediation effect value was 0.081, accounting for 45.3% (0.081/0.179) of the total effect.

### 3.3 Moderated mediation effect analysis

Model 8 was employed to determine how physical activity moderates the mediation effect among physical literacy, resilience, and mental health after controlling the influences of sex and age factors (see Table 3). The findings indicated that the effect of physical literacy × physical activity on resilience was significant (*B* = 0.001, *t* = 2.474, *p* < 0.05) but not on mental health (*B* = 0.001, *t* = 1.742, *p* > 0.05), suggesting that physical activity serves as a moderator in the prediction of physical literacy on resilience but not in the prediction of mental health.

**Table 3:** Moderated mediation effect analysis of the relationship between physical literacy and mental health.

**Regression equation**		**β**	***t***	***R***	***R^2^***	***F***
**Outcome variables**	**Predictor variables**					
RE	Sex	−0.244	−5.460^**^	0.527	0.278	77.889^**^
	Age	0.004	0.288			
	PL	0.178	16.980^**^			
	PA	−0.002	−1.218			
	PL × PA	0.001	2.474^*^			
MH	Sex	−0.003	−0.043	0.495	0.245	54.587^**^
	Age	−0.019	−1.122			
	PL	0.122	7.405^**^			
	PA	−0.008	−4.408^**^			
	RE	0.468	10.720^**^			
	PL × PA	0.001	1.742			

PL, physical literacy; PA, physical activity; RE, resilience; MH, mental health. ^*^p < 0.05, ^**^p < 0.001.

**Figure 2:**
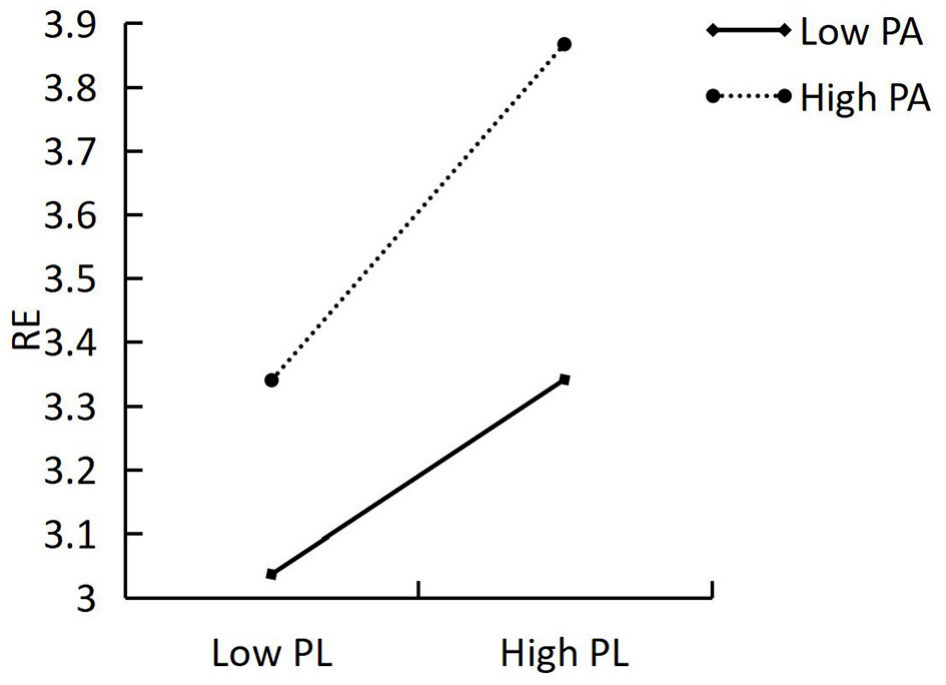
The moderating effect of physical activity on the relationship between physical literacy and resilience.

To further analyze how physical activity serves as a moderator in the relationship between mental health and physical literacy, physical activity was categorized into high and low groups based on SD criteria, and a simple slope test was performed. The results indicated that the prediction of physical literacy on resilience was significant among individuals with either high (M + 1SD, *b* = 0.197, *t* = 13.798, *p* < 0.001) or low (M – 1SD, *b* = 0.156, *t* = 12.527, *p* < 0.001) physical activity scores (Figure 2). Additionally, the predictive effect was more pronounced among individuals with high scores than among those with low scores, indicating that physical literacy influenced the prediction of resilience at higher physical activity levels.

## 4 Discussion

The present study aimed to ascertain whether resilience acts as a mediator in the relationship between physical literacy and mental health among adolescents with different physical activity levels (moderating variable) after controlling for sex and age.

The relationship between adolescents' physical literacy, physical activity, resilience, and mental health was examined and the results indicated that physical literacy, resilience, and mental health were significantly associated, even after adjusting for sex and age, which was consistent with the results of Ma et al. (2021). Furthermore, physical activity was significantly positively associated with physical literacy and resilience but not with mental health. The benefits of physical activity for mental health are well-established, and numerous studies have verified a positive relationship between them (DeJonge et al., 2020; Fluetsch et al., 2019; White et al., 2017); this study also found a significant relationship between these factors without controlling for sex and grade. Halliday et al. (2019) used a cross-sectional design to examine the role of sex in the relationship between physical activity and mental health. Their findings supported a mediation model, where physical activity mediated, rather than moderated, the relationship between sex and mental health. Kim et al.'s (2012) study also found a curvilinear relationship between general mental health and physical activity, which varied according to sex, age, and physical health status. Therefore, this study concluded that the relationship between physical activity and mental health did not reach a significant level owing to controlling for sex and grade variables.

Given that this study's results suggested that adolescents who possess greater physical literacy generally exhibit higher resilience, the findings support H1. Physical literacy can enhance the development of resilience among adolescents. These findings align with those of previous studies (Jefferies et al., 2019). Development of the confidence and motivation domains of physical literacy can enable adolescents to acquire skills and abilities and navigate different environments to maintain their wellbeing (Jefferies et al., 2019). Additionally, the positive challenges faced in developing physical competence may also position physical literacy as an antecedent of resilience (Ma et al., 2021).

Our findings align with H2, indicating that resilience has a significantly positive influence on mental health, which corroborates previous studies (O'Connor et al., 2021). Strengthening adolescents' resilience is crucial for achieving sustained mental health outcomes (Anderson and Priebe, 2021). Resilience refers to “the capacity of a dynamic system to withstand or recover from significant challenges that threaten its stability, viability, and development” (Masten, 2011). Individuals possessing higher levels of resilience can effectively and expediently recover from setbacks and challenges (Meng et al., 2023). Individuals with high resilience scores can utilize resources and dynamically adapt to changing conditions, thereby reducing the adverse effects of social restrictions on their work and life (Ho et al., 2015).

The study also supports (H3a) and (H3b). The findings revealed that physical literacy was a crucial indicator of mental health, demonstrating that adolescents with higher levels of physical literacy tend to enjoy better mental health. Physical literacy is a valuable ability closely related to human flourishing, and its association with mental health has been partially confirmed (Blain et al., 2021). This study confirmed physical literacy's important role in mental health among Chinese adolescents, showing that, in addition to improving physical health, physical literacy and psychological factors are inextricably connected (Whitehead, 2019). Furthermore, the findings supported the idea that resilience constitutes a potential mechanism that may partially explain how physical literacy is linked to mental health, which is consistent with Ma et al.'s (2021) findings. Resilience is a defense mechanism for those facing emotional depression after frustration and can promote mental health (Davydov et al., 2010). In other words, promoting physical literacy not only among college students but also among adolescents can be used as a method to promote resilience and improve mental health. The mediating effect of resilience may assist researchers in understanding the relationship between physical literacy and mental health among Chinese adolescents. The results of this study further illustrated that the contribution of physical literacy to health was not only to physical health but also to mental health.

To the best of our knowledge, this study was the first to examine the role of physical activity in the relationship between physical literacy, resilience, and mental health. Our observations confirmed H4. The findings indicated that the effect of physical literacy on resilience are not the same for individuals with different level of physical activity. Specifically, the predictive impact of physical literacy on resilience was significant for both individuals with high and low physical activity scores, and the predictive effect for individuals with high scores was better than that for those with low scores, indicating that the predictive effect of physical literacy on resilience increased with higher physical activity levels. This may be due to those who are more physically active, face and resolve setbacks more during physical activity participation, which could then lead to enhancing their resilience. Physical literacy serves as the foundation for lifelong participation in physical activities (Holler et al., 2021). Owing to several previous studies observing a moderate positive relationship between physical literacy and physical activity, physical literacy as a potential gateway to participation in physical activity is receiving increasing attention (Belanger et al., 2018; McKay et al., 2023). Brown et al. (2020) examined the association between children's physical literacy and their engagement in physical activity using latent profile analysis. The findings indicated that, after controlling for socioeconomic status and sex, children with high physical literacy profiles exhibited the highest participation in physical activity, followed by those with moderate physical literacy; the lowest participation was observed in the three low physical literacy profiles (Brown et al., 2020). Previous studies demonstrated that physical activity can enhance resilience (Xia et al., 2020) and that an increase in vigorous physical activity can significantly enhance resilience (Dunston et al., 2022). Highly physically active adolescents have better physical literacy (Brown et al., 2020; Öztürk et al., 2023) and resilience (Dunston et al., 2022) scores than moderately active and inactive adolescents. This study provides further evidence that physical activity moderates the relationship between resilience and physical literacy; the predictive effect of physical literacy on resilience increases with higher physical activity levels.

The beneficial impact of physical activity on mental health has largely garnered consensus and is often used as an intervention to promote mental health. However, this study's results did not confirm H5. This aligns with the findings of Wermelinger Ávila et al. (2022), who found that individuals who participated in regular physical activity exhibited greater resilience relative to those who did not; however, no significant differences were noted in mental health outcomes. However, longitudinal studies included in systematic reviews have generally reported the positive effects of physical activity on mental health. Although a recent systematic review and meta-analysis of 111 studies involving nearly three million individuals reported an association between physical activity and lower levels of depression, 35% of the studies showed that the relationship between the two was not significant (Dishman et al., 2021). These discrepancies may be associated with age, sample size, measures, and follow-up procedures (Wermelinger Ávila et al., 2022). In present study, when conducting moderated mediation analysis, controlling for sex and grade variables that may affect the relationship leads to the moderating effect of sports activities on the relationship between physical fitness and mental health not reaching a significant level. Another possible explanation is that the relationship between physical literacy and mental health may be indirect and influenced by other factors associated with physical literacy, such as resilience, leading to the dilution of the regulation of physical activity. Additionally, previous studies found that physical activity may be a mediating variable between mental health, physical literacy (Dong et al., 2023), and wellbeing (Melby et al., 2023). However, previous studies also found that moderate- to vigorous-intensity physical activity can partially influence the relationship between physical literacy and physical health but not the relationship between physical literacy and mental health (Melby et al., 2022; Tang et al., 2023). In summary, further research is required on the relationship between physical literacy, physical activity, and mental health.

## 5 Conclusion

This study was the first to examine the relationship between physical literacy, physical activity, resilience, and mental health. The results confirmed that resilience mediates the relationship between mental health and physical literacy among adolescents, and physical activity moderates the relationship between physical literacy and resilience in the mediator model. Physical literacy was more effective in promoting resilience among adolescents with high physical activity. However, in this study, the relationship between physical activity and mental health in adolescents was not significant after controlling for sex and grade variables, and physical activity did not moderate the relationship between physical literacy and resilience in the mediator model.

## Data Availability

The raw data supporting the conclusions of this article will be made available by the authors, without undue reservation.
